# Harnessing hollow Prussian blue nanozymes for efficient photothermal lithotripsy while protecting the kidneys from oxidative stress injury

**DOI:** 10.1016/j.mtbio.2025.102467

**Published:** 2025-10-24

**Authors:** Ziyu Ye, Yuan Tian, Hantian Guan, Yue Zhuo, Shoule Wang, Xiangya Luo, Hongxing Liu, Wen Zhong

**Affiliations:** a Department of Urology, The First Affiliated Hospital, Guangzhou Medical University, Guangzhou, Guangdong, China; b Guangdong Provincial Key Laboratory of Urological Diseases, Guangzhou Medical University, Guangzhou, Guangdong, China; c Guangdong Engineering Research Center of Urinary Minimally Invasive Surgery Robot and Intelligent Equipment, Guangzhou Medical University, Guangzhou, Guangdong, China; d Guangzhou Institute of Urology, Guangzhou Medical University, Guangzhou, Guangdong, China; e Department of Endocrinology, Key Laboratory of Biological Targeting Diagnosis, Therapy and Rehabilitation of Guangdong Higher Education Institutes, The Fifth Affiliated Hospital of Guangzhou Medical University, Guangzhou, Guangdong, China

**Keywords:** Hollow prussian blue nanozyme, Oxidative stress, Photothermal effect, Lithotripsy

## Abstract

Kidney stones, given the high incidence and recurrence rates, pose a critical challenge to public health. High-power laser lithotripsy may induce damage to renal tissues, while existing therapeutic drugs have limitations in relieving the damage to renal tubular epithelial cells induced by oxidative stress. This injury promotes crystal aggregation and adhesion, further exacerbating cell damage and forming a vicious cycle. To address this problem, we proposed hollow Prussian blue (HPB) nanozymes for efficient photothermal lithotripsy while protecting the kidneys from oxidative stress injury. The in vitro lithotripsy results demonstrate that the efficiency of lithotripsy could be enhanced by adhering HPB to the surface of kidney stones thanks to its photothermal effect and stability characteristic under low power 808 nm near-infrared laser. No significant renal tissue damage was observed after the procedure, indicating its safety. In vitro HPB can simulate the activities of various antioxidant enzymes, thereby scavenge free radicals and protect cells from oxidative stress damage. Meanwhile, an animal model of renal CaOx crystals induced by glyoxylate was established to explore the safety and therapeutic effect of HPB. HPB could scavenge reactive oxygen species (ROS) and attenuate oxidative stress through its excellent biocompatibility and antioxidant enzyme activity, which protects renal tubular cells, upregulates the expression of antioxidant enzymes, downregulates proteins associated with stone adhesion and renal injury, and ultimately inhibits crystal deposition. Collectively, HPB not only provides an experimental theoretical foundation for novel lithotripsy techniques but also offers new insights into kidney protection and the prevention of crystal deposition.

## Highlights

• This study synthesized a HPB nanozyme for efficient photothermal lithotripsy.
• HPB can not only perform photothermal lithotripsy, but also provide antioxidant protection for kidney tissue.
• HPB could inhibits crystal deposition and alleviates renal injury.

## Graphical abstract

**Figure:**
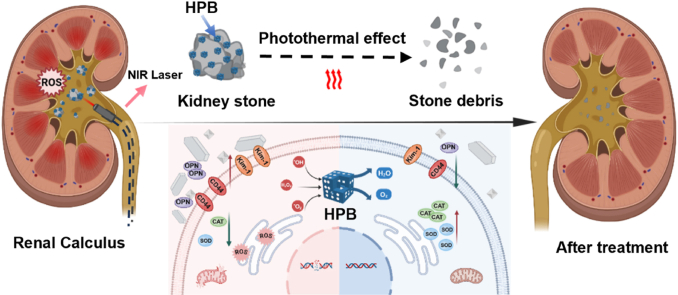

## 1 Introduction

With the increasing global incidence, kidney stones have become a highly prevalent clinical disease in urology, presenting a considerable challenge to public health. In China, kidney-stone disease impacts nearly 1 in 17 individuals [1], with half of them experiencing recurrence within 5 to 10 years [2]. Kidney stones may lead to serious complications such as urinary tract infection, low back pain, gastrointestinal reactions, hydronephrosis, and decreased renal function. The high recurrence rate further increases the need for extra surgical interventions. This not only causes physical pain and psychological distress for patients but also exerts a substantial economic strain on individuals, families, and society.

The primary surgical approaches for renal calculi involve holmium laser lithotripsy, a well-developed technique under percutaneous nephrolithotomy (PCNL) or flexible ureteroscopy (fURS). Holmium: YAG lasers fragment urinary stones primarily through photothermal ablation and photomechanical mechanisms [3]. For the sake of the increasing temperature near the tip, the laser must contact the stone closely to reduce surrounding tissue damage intraoperatively [4]. However, high-power laser settings generate significant thermal effects, which may accidently injure the renal pelvis or calyx mucosa, leading to intraoperative bleeding [5]. The temperature generated and the thermal dose delivered are higher near the tip of the laser fiber [6]. This risk increases in cases with limited anatomical space, inadequate irrigation, or prolonged procedure time [7]. Additionally, cavitation bubbles formed due to water absorption of laser energy might cause potential physical damage to renal tissue [8].

The deposition of CaOx crystals involves complex pathological processes [9]. Oxidative stress injury to renal epithelial cells is considered a primary pathological factor in stone formation [10]. The overproduction of reactive oxygen species or reduction of antioxidants breaks the balance between ROS production and scavenging, resulting in oxidative stress, inflammation and injury [11]. The renal tubules encompass abundant mitochondria to meet the high energy demands of reabsorption processes [12]. When adhered to renal tubular epithelial cell surfaces, calcium oxalate crystals undergo endocytosis and are subsequently decomposed into free calcium and oxalate ions [13]. On the one hand, these elevated ion concentrations activate NADPH oxidase through the renin-angiotensin system, generating ROS in renal epithelial cells [14]. On the other hand, calcium overload may lead to mitochondrial dysfunction, triggering excessive ROS production that ultimately blocks DNA replication [15,16]. Ultimately, it leads to cell cycle arrest and the onset of apoptosis [17]. Renal tubular epithelial cell damage further aggravates stone adhesion [18,19]. Therefore, the study of drugs that attenuate oxidative stress damage is essential to the management and treatment of kidney stones.

Despite the significant advances in medicine, limitations persist in antioxidant. Natural antioxidant enzymes are unstable, which makes them prone to digestion and requires complex synthesis under strict environmental conditions. With the development of nanomedicine, various novel nanomaterials have been employed for the therapeutic interventions of renal calculi [19,20]. Possessing the ability of catalytic, nanozymes can be fabricated in a simple and low-cost way and can be modified to meet the specific needs by altering their size, shape, or surface charge [21]. Self-assembled nanomaterials and cerium oxide (CeO_2_) nanoparticles have been explored for kidney stone therapy by alleviating oxidative stress [10,22]. However, the lack of photothermal properties limits their ability to simultaneously provide antioxidative protection and achieve lithotripsy. In parallel, current studies have utilized carbon-based and gold-based nanomaterials to pretreat the stones in order to fragment them combined with the use of near-infrared laser [23]. Nevertheless, these nanomaterials have limitations, as they are not easily degradable and fail to protect against oxidative stress.

Prussian blue is approved by the FDA as an antidote for cesium and thallium poisoning [24]. Prussian blue nanozymes (PB) possess the ability to mimic peroxidase, catalase, and superoxide dismutase enzymes [25]. Compared with PB, hollow Prussian blue nanozymes (HPB) offer a larger specific surface area, providing more reactive sites [26]. High-loading rapamycin-incorporated hollow mesoporous Prussian blue nanozymes have demonstrated targeted delivery to spinal cord injury sites, facilitating motor function recovery, thereby highlighting their effective antioxidative and anti-apoptotic capabilities [27]. The wavelength range of 700–1100 nm represents the optimal transmission window for human tissues and blood, with minimal absorption by biological tissues [28]. HPB exhibits a significant photothermal effect, efficiently absorbing 808 nm laser irradiation and converting it into thermal energy [29]. The high photothermal conversion efficiency of HPB originates from their cubic structure and the oscillation of free electrons between Fe^2+^ and Fe^3+^ [30]. As a result, HPB is widely used in photothermal therapy for tumors and in ultrasound imaging applications [31,32].

**Scheme 1:**
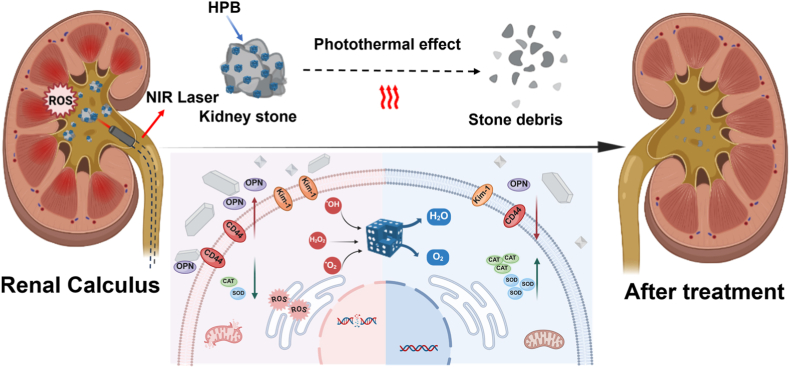
Schematic diagram for the mechanism of HPB relieving oxidative stress and the process of photonic lithotripsy.

Hence, this research aimed to develop a multifunctional nanozyme with good biosafety that enhances the efficiency of stone fragmentation combining with near-infrared (NIR) laser, inhibits the deposition of crystals, and protects renal tubular epithelial cells through anti-oxidative stress. As shown in Scheme 1, when HPB attaches to calcium oxalate stone surfaces, non-contact irradiation with an 808 nm NIR laser can fragment stones via photothermal effects. More significantly, by mimicking antioxidant enzyme activity, HPB demonstrated intracellular ROS scavenging capability while reducing apoptosis. Mechanistically, it upregulates expression of antioxidant enzymes (CAT and SOD), while downregulating stone adhesion-related molecules (CD44, OPN) and renal injury factor (Kim-1), thereby inhibiting crystal deposition. Beyond these findings, this study not only elucidates the mechanisms of nanozyme-mediated renal protection and crystal inhibition but also proposes a clinical framework for developing a novel lithotripsy approach.

## 2 Materials and methods

### 2.1 Materials

The human proximal tubular epithelial cell line (HK-2) used in experiments was purchased from the American Type Culture Collection (ATCC). Hematoxylin-eosin (HE) staining kit (G1120), anhydrous oxalic acid (SO8180) and polyvinylpyrrolidone (P8060) were obtained from Solarbio (Beijing, China). 2,2-Diphenyl-1-picrylhydrazyl (D807297) was obtained from Macklin (Shanghai, China). Cell Counting Kit-8 (CCK-8) assay solution (EK-5103) and tissue fixation solution (ES-8101) were procured from ETA (Guangzhou, China). Potassium ferricyanide (P111567) was acquired from Aladdin (Shanghai, China). Total superoxide dismutase (SOD) activity assay kit (S0101M) and Calcein/PI cell viability and cytotoxicity assay kit (C2015S) were purchased from Beyotime (Shanghai, China). Absolute ethanol (HB15-GR-0.5L) and xylene (IC02-AR-0.5L) were supplied by Guangzhou Chemical Reagent Factory.

### 2.2 Preparation and characterization of HPB

Prussian blue (PB) nanozymes were synthesized according to previously reported methods [33]. Briefly, 132 mg of K_3_[Fe(CN)_6_] and 3 g of polyvinylpyrrolidone were dissolved in 40 mL of 0.01 mol/L hydrochloric acid (HCl) solution, followed by stirring at room temperature for 30 min and subsequent reaction at 80 °C for 20 h. After centrifugation and repeated washing, the product was dried to yield PB. Subsequently, 20 mg of thoroughly dried PB and 80 mg of PVP were dissolved in 20 mL of 1 mol/L HCl solution, stirred at room temperature for 2 h, and poured into a reaction autoclave for heating at 140 °C for 2 h. The final product was collected and processed via centrifugation and washing as described above, and finally yielded 20 mg of hollow Prussian blue (HPB) after drying. The morphology and structure of PB and HPB were examined using transmission electron microscopy (TEM). EDS mapping characterized elemental composition and distribution, while XPS analysis resolved chemical states of constituent elements. 1 mg/mL ultrasonically homogenized HPB was placed into a 1 cm quartz cuvette. Set the wavelength range from 400 to 1000 nm. Then, use a UV–Visible spectrophotometer to detect the ultraviolet absorption spectrum of HPB.

### 2.3 Detection of antioxidant enzyme activity

The catalytic effect of HPB was evaluated by determining the oxygen production from hydrogen peroxide decomposition. A 6-well plate was prepared, with distilled water and HPB at concentrations of 40 μg/mL, 60 μg/mL, and 80 μg/mL added to separate wells. Subsequently, we added 20 mmol/L hydrogen peroxide solution to each well. The dissolved oxygen concentration in the reaction system was measured at 20-s intervals using a dissolved oxygen meter.

The superoxide dismutase (SOD) activity of HPB mimicking was determined using a commercial assay kit. According to the protocol, SOD assay buffer, WST-8/enzyme working solution and reaction initiation working solution were added to 96-well plates. The absorbance was measured at 450 nm after incubation at 37 °C for 30 min. Three independent replicates were performed for each experimental condition and statistical comparisons were conducted using one-way analysis of variance (ANOVA).

### 2.4 Detection of the ability to scavenge free radicals

1 mL of the 0.1 mmol/L DPPH solution was mixed with 1 mL of the HPB solution diluted with methanol. After thorough vortex mixing in the dark and 30-min incubation at room temperature, the mixture's absorbance was measured at 517 nm.

### 2.5 Detection of photothermal effect and photothermal stability

Samples with different treatments were added to 1.5 mL centrifuge tubes. The control group was treated with 600 μL of pure water, while the other five groups were treated with 600 μL of HPB solution (25, 50, 100, 200, and 400 μg/mL) diluted in pure water. The tubes, laser emitter, and thermal imager were fixed as described above. Continuous irradiation was performed at 1.0 W for 10 min and the temperature of the samples was recorded.

Add 600 μL of 500 μg/mL HPB solution to the centrifuge tube. The sample was subjected to 1.0 W laser irradiation for 10 min, followed by a 10-min laser-off interval. This cycle was repeated until the 120-min time point, with temperature recorded at 1-min intervals.

The 808 nm laser transmitter was fixed above the test tube, and the thermal imager was fixed in front of the test tube holder so that the image of the centrifuge tube was located in the center of the camera aperture. The thermal imaging picture was taken and the temperature was recorded. Finally, the temperature data was plotted as a graph.

The photothermal conversion efficiency (η) was calculated according to the following formula:

$$\eta =\frac{hS\left(T_{\max}-T_{surr}\right)-Q_{dis}}{I\left(1-10^{{-A}_{\lambda }}\right)}$$

where h S is the heat transfer coefficient, S is the surface area of the container, $T_{\max}$ is the maximum temperature of the solution, $T_{surr}$ is the ambient temperature, $Q_{dis}$ is the heat dissipated by the solvent and container, I is the incident laser power, and $A_{\lambda }$ is the absorbance of the nanomaterial at the excitation wavelength (808 nm).

### 2.6 Human kidney stone comminution with HPB in vitro

We collected stone specimens from clinical surgeries and performed compositional analyses. Those primarily composed of calcium oxalate monohydrate were selected as the study samples. Stones of similar size were selected and placed in simulated artificial urine overnight for rehydration. The stones were divided into two groups; one group was left untreated and the other group had HPB solution dripped onto the stone surface. We fixed the laser emitter (Changchun Rayliu Optoelectronic Technology Co., Ltd.) in a holder so that it was positioned 2 cm vertically above the irradiation target, and placed the thermal imager (Fluke, Tis20+) approximately 10 cm in front of the sample to perform continuous temperature recording and imaging.

### 2.7 Investigation on the effect and safety of lithotripsy on the porcine kidney model

The stone was pretreated as described above, and the stone was placed at the tip of the renal papilla of a fresh porcine kidney to simulate a situation of in vivo lithotripsy. At the end of lithotripsy, the renal papilla tissue was excised, and paraffin sections were made in transverse and longitudinal sections, respectively, and HE staining was performed to observe the tissue damage.

### 2.8 Cell culture

HK-2 cells were cultured using DMEM/F12 cell culture medium supplemented with 10 % FBS, and triple antibiotic or mycoplasma scavenger could be added to prevent contamination as appropriate. Cells were passaged at 80 % confluency and incubated at 37 °C with 5 % CO_2_. Cell activity detection: Cells were inoculated in 96-well plates and cultured for 24 h. 100 μL of HPB was added to observe the effect on cell activity; 1.25 mmol/L oxalic acid and different concentrations of HPB were added simultaneously to each well in order to assess its protective effect on injured cells. After different treatments, the cells were incubated for 24 h. After that, CCK-8 solution was added and incubated for 2 h, and the absorbance at 450 nm was measured to assess cell activity.

### 2.9 The observation of cell morphology under the microscope

HK-2 cells were seeded into 6-well plates at a density of 2 × 10^5^. The cells were divided into three groups: (i) normal group, cells were cultured with serum-free culture medium; (ii) modeling group, adding serum-free culture medium containing 1.25 mmol/L oxalic acid; and (iii) HPB-treated group, using 1.25 mmol/L oxalic acid modeling along with the addition of low and high concentrations of HPB (1.56–6.25 μg/mL) for protection treatment. Cell density and cell morphology were observed using a microscope after 24 h of culture to assess cell activity.

### 2.10 Fluorescence detection of ROS

Cells cultured in 6-well plates were loaded with 1 mL of 10 μmol/L DCFH-DA fluorescent probe and incubated at 37 °C for 20 min in the dark. After washing for three times, 1 ml of fresh serum-free culture medium was added to each well. Then we observed and photographed the cells under a fluorescence microscope immediately.

### 2.11 Calcein AM/PI staining for cell viability detection

We prepared the 6-well plates seeded with HK-2 cells and the working solution according to the instructions of the kit (Beyotime, C2015L). Following the addition of 1 mL working solution per well, the plates were incubated at 37 °C in the dark for 30 min. After incubation, the cells were observed under a fluorescence microscope (green fluorescence for Calcein AM, and red fluorescence for PI).

### 2.12 Establishment of a mouse model of calcium oxalate kidney stones

C57BL/6 male mice weighing about 20 g were randomly divided into 4 groups of 5 mice each, as follows: (1) control group (Control): 100 μL of saline was injected intraperitoneally every day; (2) modeling group (GA): each mouse was injected with glyoxylate at a dose of 65 mg/kg/d for 7 days. (3) Low concentration HPB treatment group (GA + LHPB): injection of glyoxylate as in the modeling group, and injection of 10 mg/kg/d of HPB solution on days 2, 4 and 6; (4) High concentration HPB treatment group (GA + HHPB): modeling as described above, and injection of 20 mg/kg/d of HPB solution on days 2, 4 and 6. The mice were sacrificed at day 8, the kidneys, livers, lungs, hearts and spleens were harvested and made into sections. We observed significant crystal deposition in HE-stained kidney sections under a polarized light microscope, confirming the successful establishment of the kidney stone model.

### 2.13 Hematoxylin and eosin (HE) staining

The paraffin sections were sequentially immersed into xylene I, xylene II, xylene III anhydrous ethanol I, anhydrous ethanol II, 95 % alcohol and 85 % alcohol for 5min respectively, and rinsed under running water. The slices were subsequently placed in hematoxylin staining solution for 1–2 min, washed with water, and then transferred to a blue return solution. And they were put into blue return solution, washed with water, and placed into eosin staining solution for 2–3min. After the completion of staining, the slices were each put into anhydrous ethanol, anhydrous ethanol II, anhydrous ethanol III, xylene I, and xylene II, and then sealed by the neutral tree glue. The slices were sealed using neutral gum. Finally, the section images were uploaded to computer software using a section scanner for processing.

### 2.14 Observation of renal crystal using polarized light microscopy

Open the microscope, adjust the microscope to the polarization gear; adjust the black balance, exposure time and other parameters in the computer WZ Camera software to make the crystals bright and clear, and then select the field of view to be photographed. Use the “Split and Count” function in the WZ Camera software to count the number of crystals and the percentage of area.

### 2.15 Immunohistochemistry (IHC)

After dewaxing and rehydration, sections underwent antigen retrieval (10 mM citrate, pH 6.0, 95 °C, 10 min), peroxidase block (3 % H_2_O_2_, 10 min) and BSA blocking (5 %, 1 h). Primary antibody, HRP-secondary and SABC were applied sequentially; DAB color development was timed. Sections were counterstained with hematoxylin (3 s), blued in tap water (1 h), dehydrated, cleared and mounted. Images were acquired by a scanner.

### 2.16 Hemolytic test of HPB

We collected fresh anticoagulated whole blood from mice via the retro-orbital venous plexus, and obtained pure red blood cells (RBCs) by repeated centrifugation and washing with physiological saline. The RBCs were then co-incubated with suspensions of HPB (0.38, 1.56, 3.13, 6.25, 12.5, and 25 μg/mL) to systematically investigate the hemolytic effect of HPB in a concentration-dependent manner. PBS and deionized water were used as the negative and positive controls, respectively. All samples were incubated at 37 °C for 4 h to simulate the in vivo circulatory environment. After incubation, the mixtures were centrifuged at 3000 rpm for 15 min, and the supernatant was carefully transferred to a 96-well plate. The absorbance of hemoglobin was measured at 540 nm using a microplate reader. The hemolysis ratio of HPB on RBCs at different concentrations was accurately calculated by comparing the absorbance values of each test group with those of the negative and positive controls, thereby evaluating their hemocompatibility.

### 2.17 Prussian blue staining

To verify the distribution and accumulation of hollow Prussian blue nanoparticles (HPB) in the kidney, Prussian blue iron staining was performed on renal tissue sections. First, kidney specimens were fixed, paraffin-embedded, and sectioned, followed by conventional deparaffinization and rehydration. Tissue sections were then incubated in a freshly prepared mixture of equal volumes of potassium ferrocyanide solution and hydrochloric acid, protected from light at room temperature for approximately 30 min. During this process, if iron ions released from HPB were present in the tissue, they reacted to form insoluble blue ferric ferrocyanide precipitates. The reaction was stopped by gentle rinsing with distilled water, and nuclear fast red was applied for nuclear counterstaining. Following dehydration, clearing, and mounting with neutral resin, blue iron deposits were visualized in specific renal structures, with nuclei stained red under light microscopy.

### 2.18 Biodistribution analysis by fluorescence imaging

Firstly, ICG-labeled HPB was prepared by mixing 5 mL of HPB solution (1 mg/mL) with 1 mL of ICG solution (1 mg/mL) and stirring overnight under light-protected conditions. After centrifugation, the supernatant was removed, and the precipitate was washed three times with purified water before being resuspended. Ten nude mice were then intravenously injected via the tail vein with 100 μL of the ICG-labeled HPB suspension, while saline was administered at 0 h as the control. To monitor the biodistribution, in vivo fluorescence imaging was performed under isoflurane anesthesia with an excitation wavelength of 785 nm at 0, 1, 2, 4, 10, 18, 20, 42, 64, and 72 h after injection. Finally, the mice were sacrificed after the last imaging session, and the major organs (heart, liver, spleen, lungs, and kidneys) were collected for ex vivo fluorescence imaging.

### 2.19 Statistics

The data of this experiment were statistically analyzed using Graphpad Prism and Excel. One-way ANOVA was employed for the analysis among multiple groups. The data results were presented in the form of mean ± standard deviation (mean ± SD), and P < 0.05 indicated that the difference was statistically significant. ∗∗ indicates *P* < 0.01, ∗∗∗ stands for *P* < 0.001, and ∗∗∗∗represents *P* < 0.0001.

## 3 Results and discussion

### 3.1 Preparation, characterization and enzymatic activity of HPB

**Fig. 1:**
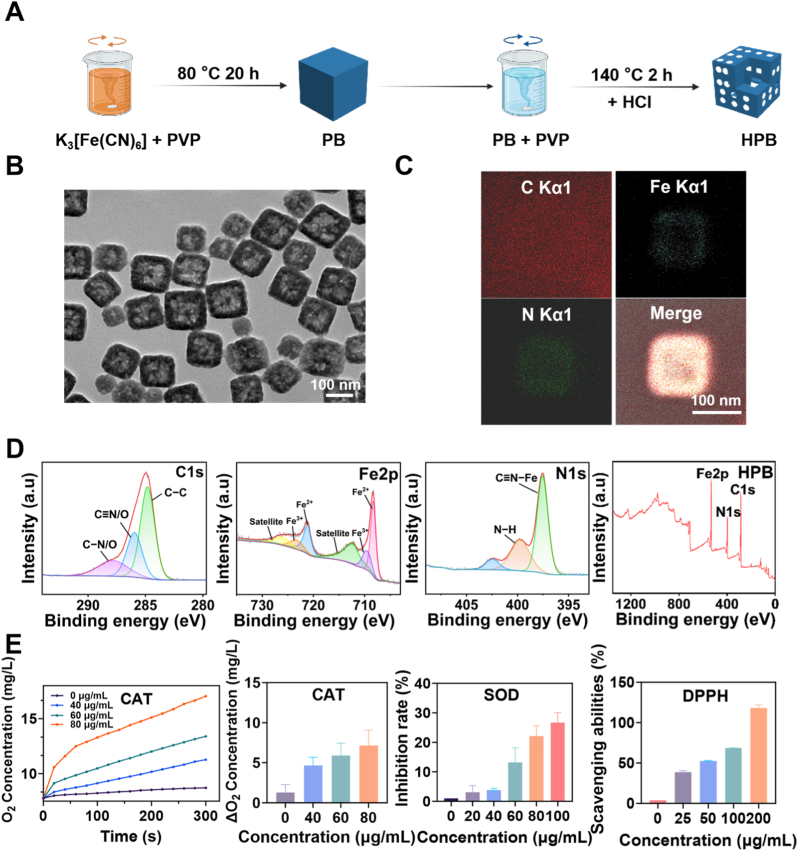
**Synthesis and characterization of HPB.** (A) Schematic illustration of the preparation of HPB. (B) Transmission electron microscopy (TEM) image of HPB, Scale bar: 100 nm. (C)Elemental mapping analysis of HPB, Scale bar: 100 nm. (D) Overall, C1s, N1s, Fe2p XPS spectra of HPB. (E) Assessment of CAT, SOD, and DPPH radical scavenging activities.

Firstly, this study optimized the synthesis conditions to fabricate HPB based on previous research [33]. The TEM images (Fig. S1) and XRD pattern (Fig. S2) of the obtained Prussian blue nanozymes is consistent with those reported in previous studies [34,35], confirming the successful synthesis of Prussian blue. Following the successful synthesis of solid Prussian blue nanozyme, HPB were prepared via hydrochloric acid etching (Fig. 1A). As shown in Fig. 1B, the image captured by transmission electron microscopy (TEM) revealed that HPB are monodisperse nanoparticles with a hollow cubic structure, where the dark regions represented the outer shell. The particles demonstrated a uniform size distribution with an average diameter of 100 nm. Energy-dispersive X-ray spectroscopy (EDS) analysis of the elemental composition and distribution confirmed that HPB consisted of C, Fe, and N. The merged elemental mapping demonstrated excellent overlap of these elements, which together formed the hollow cubic structure (Fig. 1C). X-ray photoelectron spectroscopy (XPS) analysis identified characteristic peaks corresponding to Fe 2p, C 1s, and N 1s (Fig. 1D), which are consistent with the EDS findings. The zeta potential of HPB is −13.5 mV (Fig. S3). As shown in Fig. S4, HPB exhibits a strong and broad absorption peak around 700 nm. This is the characteristic absorption peak of the metal-ligand charge transfer transition between Fe^2+^-CN-Fe^3+^, which indicates that it can convert the absorbed light energy into thermal energy and release it, thereby enabling a photothermal effect. These characterization results collectively verified the successful synthesis of HPB.

A series of in vitro experiments were carried out to verify that HPB possesses mimetic enzyme activity and the ability to scavenge free radicals (Fig. 1E). Firstly, the catalytic activity of HPB mimicking catalase was evaluated by assessing its ability to decompose hydrogen peroxide (H_2_O_2_) to generate oxygen (O_2_). The amount of O_2_ produced by HPB increased over time in a concentration-dependent manner. Secondly, the SOD-like enzymatic activity of HPB at different concentrations (20, 40, 60, 80, and 100 μg/mL) was assessed in vitro using a commercial assay kit. HPB exhibited significant catalytic activity, which improves with increasing concentration. The DPPH solution was used to assess the free radical scavenging activity of HPB. Following HPB addition, a reduction in absorbance was observed. The absorbance values were converted to free radical scavenging rates using standard formulas, demonstrating concentration-dependent enhancement of radical scavenging capacity. HPB exhibits enzyme-like activity due to its mixed-valence Fe^2+^/Fe^3+^ structure, which enables cyclic redox reactions to scavenge reactive oxygen species. Its hollow architecture further enhances this catalytic efficiency by providing more active sites. These findings can be attributed to the unique crystal structure and redox properties of HPB.

**Fig. 2:**
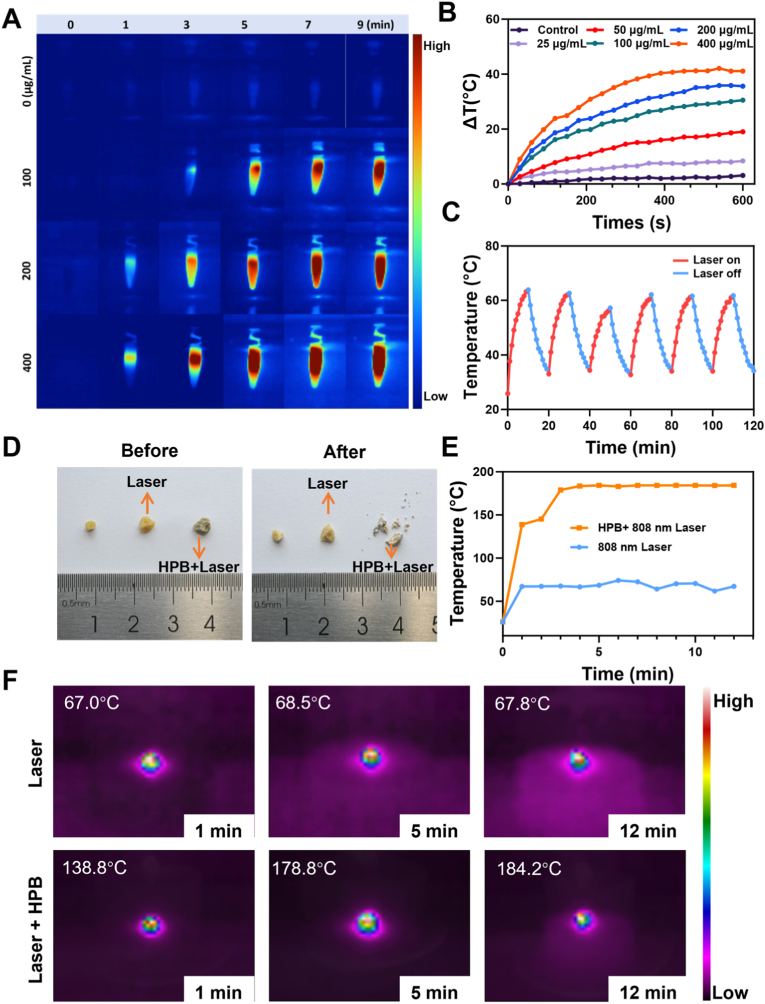
**Photothermal effect and photonic lithotripsy in vitro. (A)** Thermal imaging of water and HPB solutions at different concentrations (100, 200 and 400 μg/mL) after 808 nm laser irradiation. The color transition from blue to red in the thermal imaging scale corresponds to a progressive rise in the solution's temperature. **(B)** Temperature changes (ΔT, °C) of water and HPB solutions at different concentrations (25, 50, 100, 200 and 400 μg/mL), (ΔT = temperature at the given time point minus initial temperature). **(C)** Temperature changes of the solution during on/off cycles of laser irradiation. HPB solution was exposed to 1.0 W laser for 10 min, followed by 10-min laser-off intervals. **(D)** In vitro near-infrared laser fragmentation process of a calcium oxalate stone. The first stone was untreated, while the second and third stones were irradiated with a laser for 12 min. **(E)** Recorded temperature during the stone fragmentation process. **(F)** Thermal images of the stone fragmentation process for the laser-only group (Laser) and the HPB-treated plus laser group (Laser + HPB). The temperature labeled in the figure represents the highest central temperature. (For interpretation of the references to color in this figure legend, the reader is referred to the Web version of this article.)

To evaluate the hemolytic effect of HPB, we conducted a hemolysis experiment of HPB. As displayed in Fig. S5, the experimental results indicated that at the concentration of 0.38 – 25 μg/mL, HPB did not exhibit any visible hemolysis phenomenon, which suggests that HPB has good biological safety.

### 3.2 Photothermal effect and lithotripsy

To evaluate the stability and capability of HPB in converting near-infrared light into thermal energy, HPB solutions at different concentrations were irradiated with a 1.0 W laser. Temperature changes were continuously monitored and recorded using a thermal imaging camera. In the control group containing distilled water, no significant temperature variation was observed even after 9 min of irradiation. At an HPB concentration of 100 μg/mL, a noticeable temperature increase was detected starting from the fifth minute. When the concentration was increased to 400 μg/mL, the temperature rise was evident as early as the first minute (Fig. 2A). Fig. 2B presents the temperature changes of HPB solutions at various concentrations after laser irradiation. While the control group showed no marked temperature difference after irradiation, the solution containing 25 μg/mL HPB exhibited a temperature increase of approximately 8 °C by 600 s. HPB at a concentration of 400 μg/mL exhibited a temperature increase of up to 42.1 °C compared to the initial temperature after irradiation. The corresponding curve clearly demonstrates a significant rise in temperature with increasing HPB concentration. These results indicate that HPB possesses a photothermal effect that is dependent on its concentration.

The photothermal effects of different nanomaterials may decline after laser irradiation [36]. After confirming the photothermal effect of HPB, we further assessed its photothermal stability. A 500 μg/mL HPB solution was irradiated with an 808 nm laser for 10 min, followed by a 10 min cooling period. This cycle was repeated six times, with the temperature recorded every minute. As shown in Fig. 2C, the nanomaterials absorb light energy and convert it into heat at the initial stage of laser irradiation, causing a rapid increase in temperature. Once the laser is turned off, the temperature begins to decrease. After six cycles, the temperature of HPB remained stable, indicating that HPB possesses excellent photothermal stability. Then, the photothermal conversion efficiency (PCE) of HPB was evaluated under 808 nm laser irradiation. Based on the standard calculation formula, the PCE was determined to be 21.4 %. This result highlights the ability of HPB to perform photothermal lithotripsy.

The photothermal effect of HPB originates from metal-to-metal charge transfer between Fe^2+^ and Fe^3+^ ions through cyanide bridges, which efficiently converts light energy into heat under near-infrared irradiation. Its strong coordination bonds form a stable three-dimensional network, ensuring excellent structural and chemical stability during photothermal processes.

Subsequently, this study investigated whether pretreatment of calculi with HPB combined with 808 nm near-infrared laser irradiation could enhance lithotripsy. With approval from the Urology Laboratory of Guangzhou Medical University and informed consent from patients, human calcium oxalate calculus specimens were obtained. Fig. 2D displays three calcium oxalate calculi of similar size and morphology. The calculi were rehydrated overnight in artificial simulated urine. HPB was applied to the surface of the third stone, and both the second and third stones were irradiated at the same power. The HPB-treated calculus turned into fragments upon laser irradiation, whereas the others remained unaffected. Temperature changes during laser lithotripsy were recorded (Fig. 2E). The calculus treated solely with laser irradiation exhibited minimal temperature increase. Conversely, the HPB-pretreated calculus experienced a prompt temperature rise upon initial laser exposure, followed by a plateau phase with a relatively stable temperature. Fig. 2F presents thermal imaging of calculi subjected to different treatments. After 1 min of irradiation, the temperature of the laser-only group reached 67 °C, while that of the HPB-treated calculus reached 138.8 °C, with the central region peaking at 184.2 °C at 12 min. When HPB pre-treated stone was subjected to non-contact low-intensity (<2 W) laser irradiation, the nanomaterial was activated, transferring photothermal energy to the calculus, leading to calculi fragmentation. The fragmentation of CaOx stones by HPB can be explained by its unique structural and surface properties. Under NIR irradiation, Fe^2+^/Fe^3+^ oscillation generates localized interfacial heating, which induces thermal stress and microcracks within the stone. In addition, the interaction between HPB and Ca^2+^ facilitates nanoparticle adherence, ensuring precise delivery of photothermal energy to the stone interface.

### 3.3 Performing lithotripsy on porcine kidney model and safety verification

**Fig. 3:**
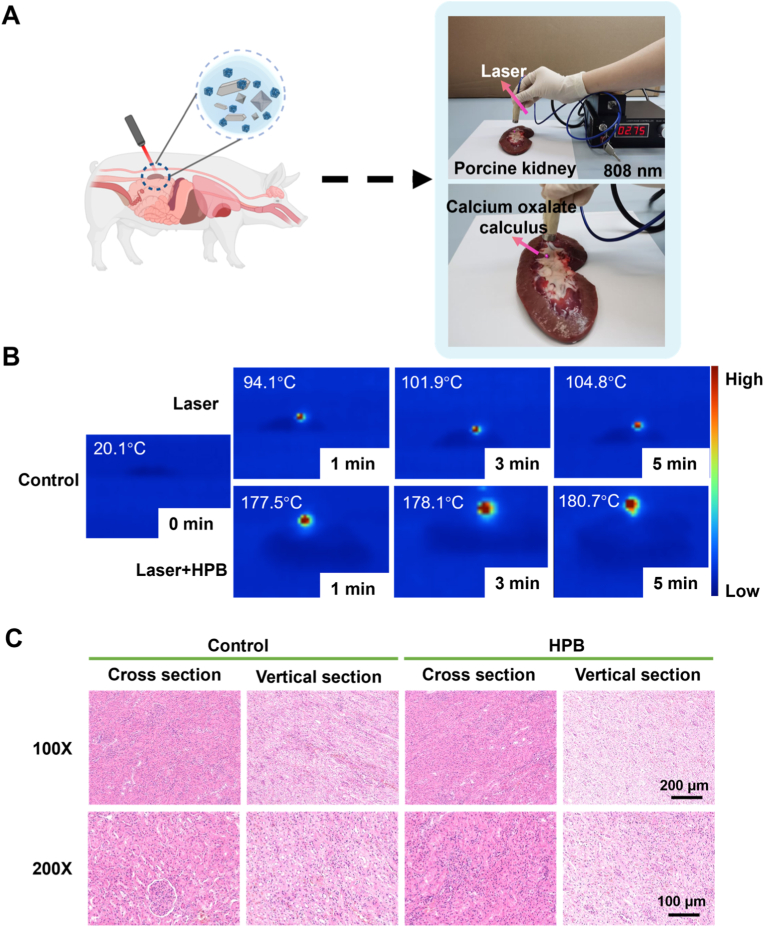
**Lithotripsy in a porcine kidney model.** (A) Schematic of laser device. A 2W, 808-nm laser was emitted from the optic fiber and directed at the calculus from a distance of 2 cm. (B) Thermal imaging of lithotripsy on a porcine kidney, with the highest central temperature of kidney stone marked in figure. (C) H&E-stained sections of kidney tissue after lithotripsy. Control group: normal kidney tissue. HPB group: a tissue where the stone which was pretreated with HPB placed on a stone. Magnification: 100×, Scale bar: 200 μm; Magnification: 200×, Scale bar: 100 μm.

After verifying the feasibility of HPB pretreatment for lithotripsy, we conducted lithotripsy using porcine kidneys in vivo. Fig. 3A illustrates the laser device and the schematic diagram of lithotripsy employed in this experiment. The right part of the figure displays an 808 nm laser emission device, where the laser was delivered through the fiber tip at a current of 2.75 A and a power of 2.0 W, with the fiber positioned 2 cm from the calculus. The calculus was placed near the renal papilla within the renal pelvis and exposed to laser irradiation. Thermal imaging monitored temperature changes during lithotripsy (Fig. 3B). The initial temperature of the stone on the porcine kidney was 20.1 °C. After 5 min of laser irradiation alone, the temperature reached 104.8 °C. In contrast, when HPB was applied to the calculus surface, the temperature rapidly rose to 177.5 °C within the first minute of irradiation. These results demonstrate the simulated renal lithotripsy scenario and the corresponding temperature dynamics.

Afterward, we further evaluated the safety of the nanomaterials combined with NIR laser lithotripsy. Kidney tissue from the lithotripsy site and normal kidney tissue were resected for comparison after the procedure. Then, both the cross-section and longitudinal section of the kidney were stained to observe the tissue structure and cellular morphology. In the HPB group, renal glomeruli appeared round with well-defined contours, showing no abnormalities such as atrophy, hypertrophy, or irregular morphology. Cells maintained tightly aligned, and no significant pathological alterations were observed in the tissue (Fig. 3C). These results indicate that pretreating renal calculi with HPB combined with near-infrared laser for lithotripsy is a safe and reliable approach that does not result in obvious damage to local tissues.

Although HPB showed promising photothermal and ROS-scavenging effects, the current validation of its stone-fragmentation efficacy has only been validated in vitro porcine kidney experiments and requires further verification. Further in vivo studies using large-animal models (such as rabbits or pigs) are needed to confirm its lithotripsy potential to optimize its dosage and safety for clinical translation.

### 3.4 HPB protects HK-2 cells by reduce oxidative stress

**Fig. 4:**
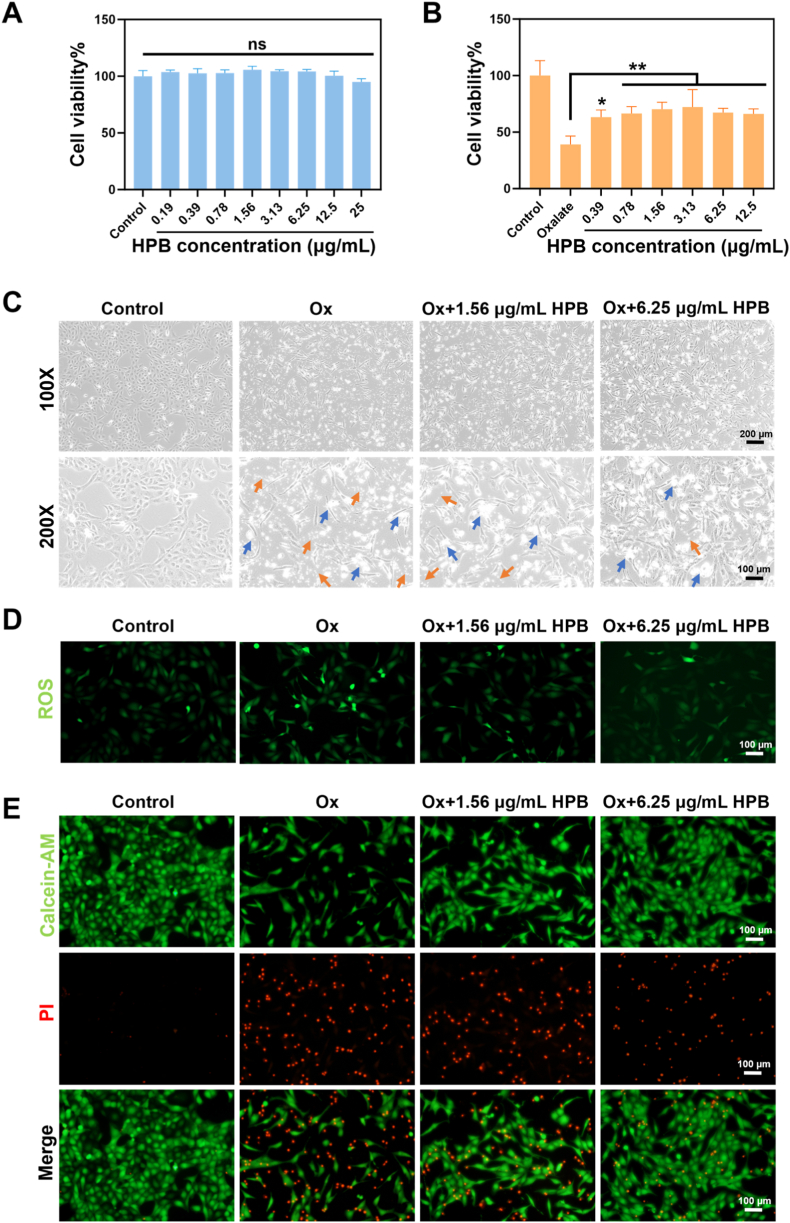
**Protective effect of HPB against oxalate-induced damage in HK-2 cells.** (A) The effect of various HPB concentrations (0.19, 0.38, 0.78, 1.56, 3.13, 6.25, 12.5 and 25 μg/mL) on cells viability. (B) The cytoprotective effect of various HPB (0.38, 0.78, 1.56, 00.313, 6.25 and 12.5 μg/mL) on damaged HK-2 cells. Oxalate concentration is 1.25 mmol/L ∗ indicates *P* < 0.05 and ∗∗ represents *P* < 0.01. (C) Cell status in culture dishes observed under optical microscope in bright field (Magnification: 100X, Scale bar: 200 μm; 200X, Scale bar: 100 μm). Oxalate concentration is 1.25 mM. HPB concentrations are 1.56 and 6.25 μg/mL. Cells were co-incubated with different solutions for 24h after plating. The blue arrows indicate cell vacuolization and apoptosis, while the orange arrows point to calcium oxalate crystals. (D) ROS fluorescence images (Magnification: 200×, Scale bar: 100 μm). Oxalate concentration is 1.25 mmol/L. HPB concentrations are 1.56 μg/mL and 6.25 μg/mL. (E) Calcein/PI fluorescence images. Green fluorescence reflects cell viability and red fluorescence represents dead cells (Magnification: 200×, Scale bar: 100 μm). (For interpretation of the references to color in this figure legend, the reader is referred to the Web version of this article.)

To assess the biosafety of HPB, HK-2 cells were incubated with graded concentrations of HPB (0.19–25 μg/mL) for 24 h and analyzed for cell viability using the CCK-8 assay. As depicted in Fig. 4A, no significant statistically differences in cell viability were observed between the HPB-treated and the control group (P > 0.05). Certain concentrations of HPB solution show no toxicity to cells, suggesting that HPB has good biocompatibility. After confirming the good biosafety of HPB, we carried out experiments to verify its protective effect on HK-2 cells modeled with oxalic acid injury within the safe concentration range (0.38, 0.78, 1.56, 3.13, 6.25 and 12.5 μg/mL). As shown in Fig. 4B, the cell viability in the oxalate-treated group dropped to around 40 % compared to the control group, but rebounded to 60 % – 70 % upon HPB treatment during the modeling period. The above data demonstrate that HPB intervention can effectively protect HK-2 cells and improve cell activity under oxalate-induced injury conditions.

Based on the above results of CCK-8 cell activity, we used 1.25 mmol/L oxalic acid for modeling. HPB at concentrations of 1.56 and 6.25 μg/mL was selected to treat the cells, respectively. Then, we observed the cell growth status under a normal light microscope (Fig. 4C). In the view of 100× magnification, the number of cells decreased after treatment with oxalate compared with the control group. The number of cells was restored with increasing concentrations of HPB.

After modeling, the cells underwent significant morphological changes. Their shape shifted from rounded to elongated with irregular morphology. Obvious vacuolization also appeared in the cytoplasm. Additionally, a great deal of the calcium oxalate monohydrate crystals aggregated around the HK-2 cells, which induced damage related to oxidative stress [22]. In the group protected by HPB, cell morphology improved and the number of crystals aggregation around the cells decreased. These results indicate that HPB at specific concentrations can improve the condition of damaged cells and attenuate the aggregation and adhesion of calcium oxalate crystals.

We investigated the antioxidant capacity of HPB by detecting intracellular ROS levels using the DCFH-DA fluorescent probe. Fig. 4D shows that the green fluorescence intensity increased significantly after the addition of oxalic acid, indicating that oxalic acid stimulated HK-2 cells to generate excessive ROS. Dose-dependent HPB addition diminished the green fluorescence gradually, approaching that of the control group, demonstrating a decrease in intracellular ROS levels. These results demonstrate that HPB can effectively scavenge intracellular ROS, thereby reducing cellular oxidative stress and exerting a cytoprotective effect.

Calcein-AM is converted by intracellular esterases into green fluorescent calcein in living cells. As a kind of cell membrane-impermeable nucleic acid dye, Propidium iodide (PI) specifically binds to DNA in dead cells and emits red fluorescence. In this experiment, cell activity and cytotoxicity were assessed using these two probes together, providing a comprehensive reflection of cell status. As shown in Fig. 4E, control HK-2 cells displayed strong green fluorescence and almost no red fluorescence. The model group exhibited reduced green fluorescence intensity and elevated red fluorescence intensity, indicating diminished cell viability and increased cell death. After HPB intervention, the green fluorescence intensity of the cells increased, while the red fluorescence intensity decreased. As shown in Fig. S6, quantified analysis revealed significant differences in cell viability across groups. The control group exhibited minimal cell death, whereas the modeling group showed a significant increase in dead cells. The addition of HPB at gradient concentrations effectively reduced cell death. These results indicate that HPB can effectively protect HK-2 cells as well as reduce cell death caused by oxalic acid injury.

### 3.5 Biocompatibility assessment of HPB in animal model

**Fig. 5:**
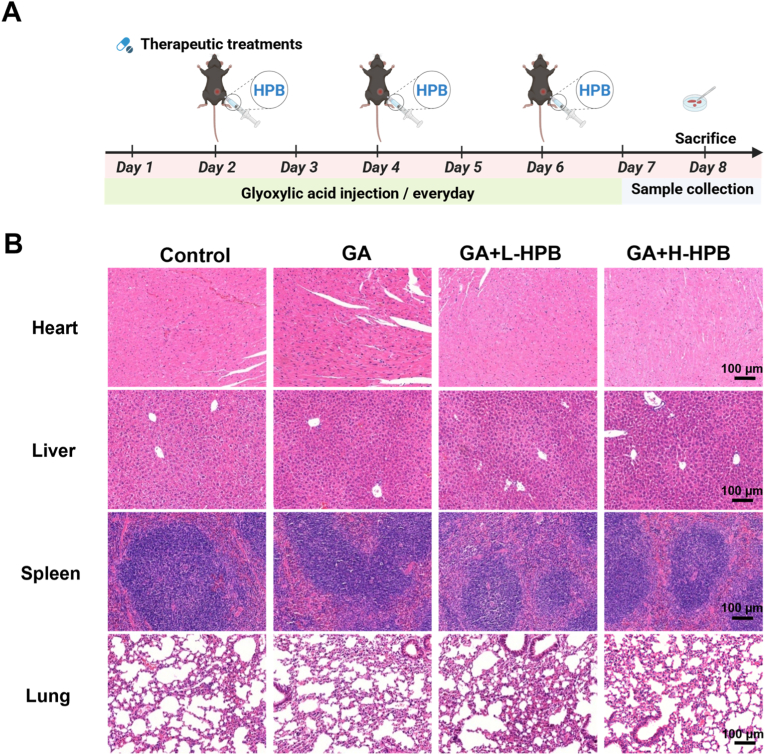
**Safety evaluation of HPB in a mouse model of kidney stones.** (A) Schematic diagram illustrating the construction of a mouse model of kidney crystal deposition using glyoxylic acid (GA). Mice were administered HPB treatment via intraperitoneal injection every other day. (B) H&E-stained sections of major organs harvested from mice in different treatment groups. Control: Control group GA: Glyoxylic acid group L-HPB: Low-dose HPB group (10 mg/kg/d) H-HPB: High-dose HPB group (20 mg/kg/d) Magnification: 200× , Scale bar: 100 μm.

To evaluate the in vivo safety of HPB and its antioxidant potential for kidney protection and stone inhibition, we established a mouse renal stone model based on a previous study [37]. The dose of glyoxylic acid was adjusted to 65 mg/kg/day to minimize potential toxicity and prevent mouse mortality, while ensuring the successful establishment of the calcium oxalate kidney stone model. HPB was administered intraperitoneally every other day at low (10 mg/kg/day) and high (20 mg/kg/day) doses. Mice were sacrificed on the 8th day (Fig. 5A). HE-stained sections of major organs (Fig. 5B) revealed no significant pathology in the heart, liver, spleen, or lungs, thus confirming HPB's in vivo safety.

Prussian blue iron staining of renal tissue sections was conducted to evaluate the distribution and accumulation of hollow Prussian blue nanoparticles (HPB) in the kidney (Fig. S7). Under microscopy, blue granular deposits were observed in the renal tissues of HPB-treated mice while no obvious blue granular deposits were found in the renal pelvis of the control group, indicating the distribution of this nanomaterial within the kidneys. These positive signals were predominantly localized in the renal pelvis, whereas no significant staining was detected in the glomeruli. These findings visually confirm that HPB reach the kidneys via systemic circulation and are subsequently excreted without inducing cumulative toxicity.

The biodistribution of HPB was investigated in vivo using small-animal imaging after intravenous injection of ICG-labeled HPB. The results demonstrated that HPB initially accumulated predominantly in the liver after injection, while a marked accumulation was observed in the kidneys at around 20 h (Fig. S8). By 72 h, HPB was almost completely metabolized, indicating its efficient renal targeting and favorable clearance without evidence of cumulative toxicity. Notably, this study employed a spatiotemporally controllable laser for photothermal lithotripsy. In future applications, the laser can be delivered directly to the intrarenal stone site via the ureter or through percutaneous nephrolithotomy. Therefore, transient retention of HPB in other organs is not expected to result in adverse effects on essential organs.

### 3.6 Kidney protection

**Fig. 6:**
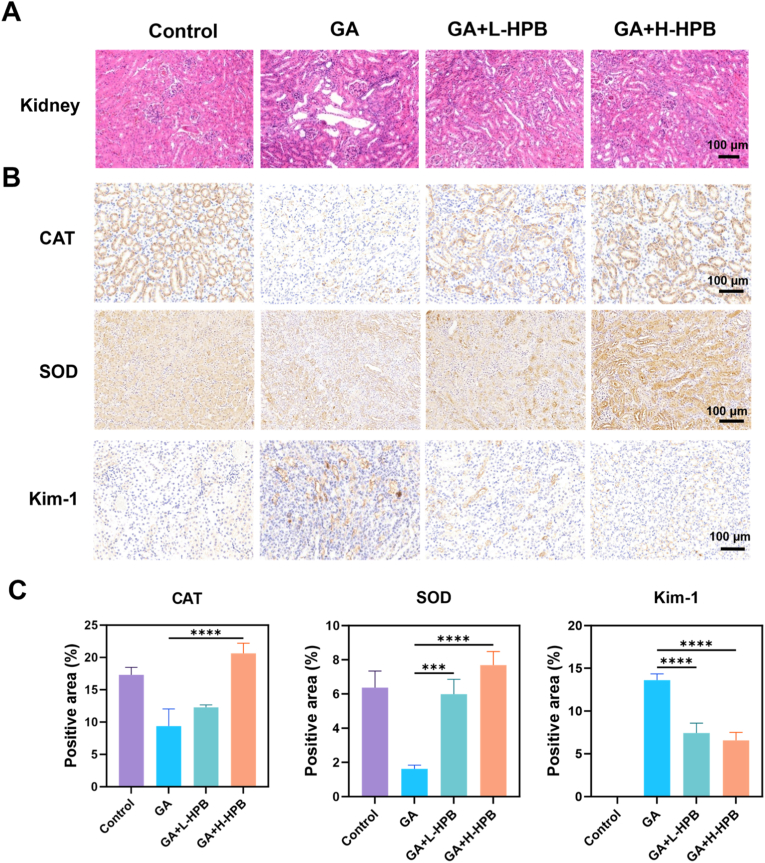
**Immunohistochemical analysis of oxidative damage-related proteins.** (A) The HE-staining of kidney tissue reflects the situation of renal injury under different treatments. (B) Immunohistochemical detection of antioxidant enzymes (CAT, SOD), adhesion molecules (OPN, CD44), and kidney injury molecule (Kim-1). Control: Control group GA: Glyoxylic acid group L-HPB: Low-dose HPB group (10 mg/kg/day) H-HPB: High-dose HPB group (20 mg/kg/day); Magnification: 200× , Scale bar: 100 μm. (C) Semi-quantitative analysis (using ImageJ software) of the expression levels of catalase (CAT), superoxide dismutase (SOD), and kidney injury molecule-1 (Kim-1). ∗∗ stands for *P* < 0.01, ∗∗∗ indicates *P* < 0.001, ∗∗∗∗ represents *P* < 0.0001.

Obvious pathological changes could be observed in the HE-stained sections of the kidney tissues of mice in the glyoxylate modeling group (Fig. 6A), with a large number of neutrophil infiltrations, tubular dilatation, glomerular atrophy, and increased cystic luminal area of the renal corpuscles.

After the intervention of HPB, renal injuries were alleviated, with a reduction in the infiltration of inflammatory cells, a minimal dilatation of the renal tubules, and no obvious atrophy of the glomeruli. Aiming to evaluate the effect of HPB on the antioxidant capacity of renal tissue and clarify its role in alleviating oxidative stress in the kidney, this study analyzed the expression levels of related proteins expression levels via immunohistochemistry (Fig. 6B). Table S1 illustrates the antibodies used in immunohistochemistry. SOD converts oxide anions (O^2−^) into hydrogen peroxide (H_2_O_2_), while CAT further decomposes H_2_O_2_ into water (H_2_O) and oxygen (O_2_) [38]. They collectively eliminate ROS and safeguard intracellular redox balance [39]. In the modelling group, CAT and SOD expression levels were significantly reduced in mouse kidney tissues, likely due to substantial antioxidant enzyme consumption from ROS production. Low concentration of HPB intervention could increase the expression level of antioxidant enzymes, while high concentration of HPB intervention could significantly restore the antioxidant capacity of the kidney.

Kim-1 is a sensitive marker for kidney injury, which is used for early diagnosis and monitoring [40]. In the glyoxylate modelling group, elevated Kim-1 levels in mouse renal tissues indicated renal injury in the renal crystal deposition model. HPB intervention at 10 mg/kg/d reduced renal injury molecule expression levels, with the best intervention effect achieved at 20 mg/kg/d HPB. Fig. 6C presents the semi-quantitative analysis of antioxidant enzymes and renal injury markers, showing a significant statistical difference between the modeling group and the HPB intervention group. The results revealed that HPB could compensate for the reduction in antioxidant enzyme levels induced by the modeling process, thereby enhancing the antioxidant defense capacity of renal tissue. This improvement contributes to a decreased expression of kidney injury markers and provides substantial protection of kidney function.

### 3.7 Reducing crystal deposition by down-regulating adhesion molecules

**Fig. 7:**
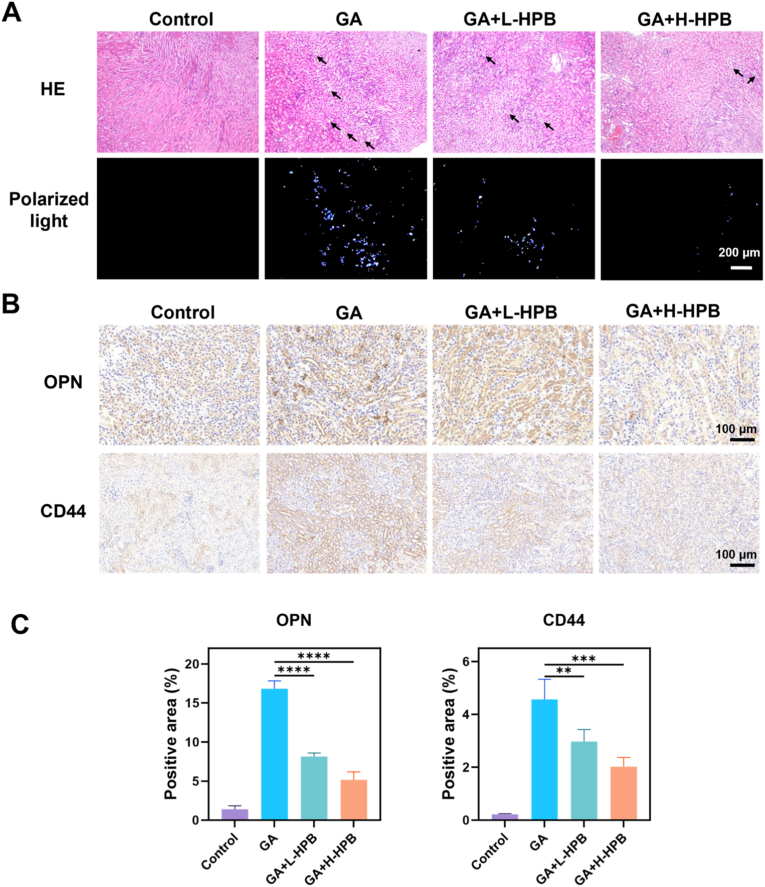
**Inhibition of crystal formation and deposition.** (A) Bright-field microscopy (upper) and polarized light analysis (lower) of HE-stained renal tissue; The black arrows point out the areas of crystal deposition. Magnification: 100× ; Scale bar: 200 μm. Control: control group; GA: glyoxylic acid; L-HPB: low concentration HPB, 10 mg/kg/day; H-HPB: high concentration HPB, 20 mg/kg/day; Magnification: 100× ; Scale bar: 200 μm. (B) Immunohistochemical detection of adhesion molecules (OPN, CD44); Magnification: 200× ; Scale bar: 100 μm. (C) Semi-quantitative analysis of osteopontin (OPN) and cell adhesion molecule CD44 expression levels using ImageJ. ∗∗ indicates *P* < 0.01; ∗∗∗ stands for *P* < 0.001; ∗∗∗∗ represents *P* < 0.0001.

We observed the crystal deposition of HE sections under ordinary microscope and polarized light microscope, respectively. Fig. 7A shows that there was no crystal accumulation in the control group. Conversely, notable crystal deposition could be observed in the modeling group, which was primarily distributed in the lumen of the renal tubules. Using polarized light microscopy, abundant crystals, which appeared as bright punctate structures, were observed in the field of view, indicating the successful construction of the animal kidney stone model. Following HPB intervention, mice were administered low-concentration HPB (10 mg/kg/d) exhibited reduced crystal deposition. More notably, intraperitoneal injection of high-concentration HPB (20 mg/kg/d) further significantly reduced renal crystal burden. The WZ Camera software was used to perform a statistical analysis of the crystal count and area in the captured images (Fig. S9). Successful establishment of the kidney stone model was confirmed by the high crystal count and the substantial crystal-covered area in the modeling group. Mice injected intraperitoneally with HPB exhibited fewer kidney crystals and a reduced crystal area compared with the modeling group. The above results suggest that HPB can reduce glyoxylate-induced kidney stone formation.

Epithelial cells of renal tubules undergo cellular changes upon exposure to urinary crystals and concurrently activate pathways related to inflammation, cell adhesion, and osteogenesis [41]. OPN promotes stone aggregation and nucleation, and CD44 is crucial in calcium oxalate crystal adhesion [42]. The intraperitoneal injection of HPB could reduce the levels of these stone adhesion-related molecules, with high dose of HPB group could significantly reduce the expression of OPN and CD44 (Fig. 7B–C). These findings indicated that HPB suppressed the expression of crystal adhesion molecules in the kidneys of mice modeled with glyoxylate, thereby inhibiting stone formation.

## 4 Conclusion

Oxidative stress is a major mechanism involved in kidney stone formation. Current drugs have limitations in antioxidant, and high-power laser lithotripsy may potentially cause damage to renal tissue. HPB possesses a photothermal conversion effect, enabling it to transduce 808 nm laser energy into heat, which enables stone fragmentation without the need for direct contact between the laser fiber tip and the calculi. Moreover, HPB demonstrates good biocompatibility and exhibits activities of multiple antioxidant enzymes. By scavenging ROS and free radicals, HPB effectively helps maintain redox balance in the renal microenvironment. This alleviates oxidative stress-induced injury to renal tubular epithelial cells caused by oxalate and crystal deposition. This reduces crystal adhesion and protects renal tissue. Therefore, HPB acts as a multifunctional nanozyme, offering a novel approach to lithotripsy and kidney protection as well as shedding light on the therapeutic strategy of urinary tract stones using nanomaterials.

## CRediT authorship contribution statement

**Ziyu Ye:** Writing – original draft, Validation, Resources, Methodology, Investigation, Formal analysis, Data curation. **Yuan Tian:** Visualization, Resources, Methodology, Investigation. **Hantian Guan:** Resources, Methodology. **Yue Zhuo:** Resources. **Shoule Wang:** Resources. **Xiangya Luo:** Visualization, Supervision, Resources. **Hongxing Liu:** Writing – review & editing, Writing – original draft, Supervision, Software, Resources, Project administration, Methodology, Investigation, Formal analysis. **Wen Zhong:** Writing – review & editing, Writing – original draft, Visualization, Validation, Supervision, Software, Resources, Project administration, Methodology, Investigation, Funding acquisition, Formal analysis, Conceptualization.

## Ethical approval

This research complied with all relevant ethical guidelines approved by the Animal Protection and Use Committee of the First Affiliated Hospital of Guangzhou Medical University. The animal experimental protocol was approved by the Experimental Animal Management and Use Committee of Guangzhou Top-Bio Biotechnology Co., Ltd. (Ethics number: LFTOP-IACUC-2023-0018). In accordance with ethical guidelines and regulatory requirements, human kidney stone specimens were obtained from the Urology Laboratory of the First Affiliated Hospital of Guangzhou Medical University (Ethics number: ES-2024-237).

## Consent for publication

All authors have reviewed and approved the final manuscript for submission to this journal.

## Funding

This research was financed by the National Natural Science Foundation of China (No. 82170777 and 82572388), Guangzhou Municipal Science and Technology (2025A03J4319) and Natural Science Foundation Project of Guangdong Province (2021A1515011119).

## Declaration of competing interest

The authors declare that they have no known competing financial interests or personal relationships that could have appeared to influence the work reported in this paper.

## Data Availability

The datasets used during the current study are available from the corresponding author on reasonable request.
