# Supplementary material for: Harnessing hollow Prussian blue nanozymes for efficient photothermal lithotripsy while protecting the kidneys from oxidative stress injury
Source: Mater Today Bio. 2025 Oct 24;35:102467. doi: 10.1016/j.mtbio.2025.102467 (PMC12637088; doi:10.1016/j.mtbio.2025.102467)
Supplement: Multimedia component 1 [file mmc1.docx]

**Supplementary Materials**

**Harnessing hollow Prussian blue nanozymes for efficient photothermal lithotripsy while protecting the kidneys from oxidative stress injury**

Ziyu Ye^1-4#^, Yuan Tian^1-4#^, Hantian Guan^1-4#^, Yue Zhuo^1-4^, Shoule Wang^1-4^, Xiangya Luo ^5*^, Hongxing Liu^1-4*^, Wen Zhong^1-4*^

1. Department of Urology, The First Affiliated Hospital, Guangzhou Medical University, Guangzhou, Guangdong, China
2. Guangdong Provincial Key Laboratory of Urological Diseases, Guangzhou Medical University, Guangzhou, Guangdong, China
3. Guangdong Engineering Research Center of Urinary Minimally Invasive Surgery Robot and Intelligent Equipment, Guangzhou Medical University, Guangzhou, Guangdong, China
4. Guangzhou Institute of Urology, Guangzhou Medical University, Guangzhou, Guangdong, China
5. Department of endocrinology, Key Laboratory of Biological Targeting Diagnosis, Therapy and Rehabilitation of Guangdong Higher Education Institutes, The Fifth Affiliated Hospital of Guangzhou Medical University, Guangzhou, Guangdong, China

Address: Kangda Road 1#, Haizhu District, Guangzhou, Guangdong, China, 510230

*Address correspondence to:

Wen Zhong: [gzgyzhongwen@163.com](mailto:gzgyzhongwen@163.com)

Hongxing Liu: [liuhongxing@gzhmu.edu.cn](mailto:liuhongxing@gzhmu.edu.cn)

Xiangya Luo: [2018687012@gzhmu.edu.cn](mailto:2018687012@gzhmu.edu.cn)

#These authors contributed equally to this work.


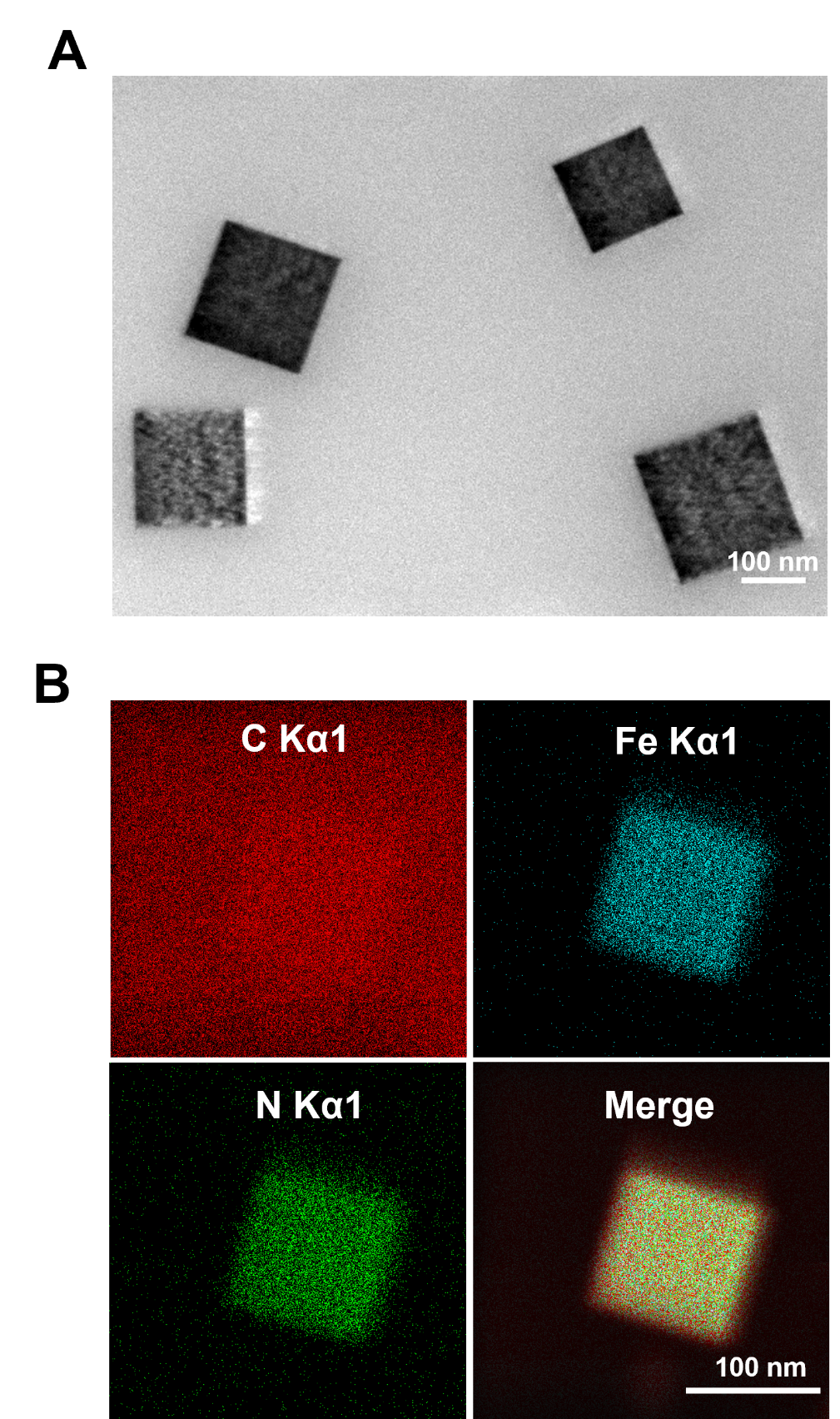


**Figure S1. Characterization of PB** (A) Transmission electron microscopy (TEM) image of PB, Scale bar: 100nm. (B) Mapping analysis of the PB (C, Fe, N). Scale bar: 100 nm.


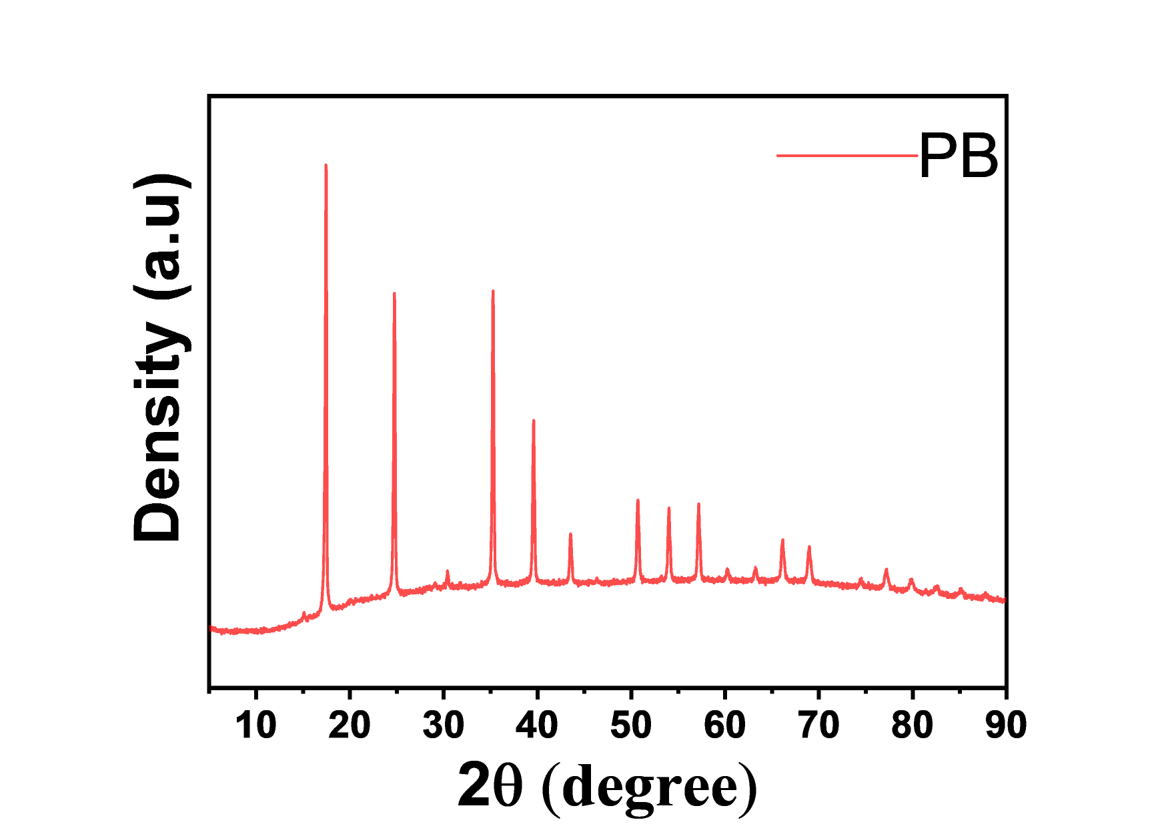


**Figure S2.** X-ray diffraction (XRD) spectra of PB.


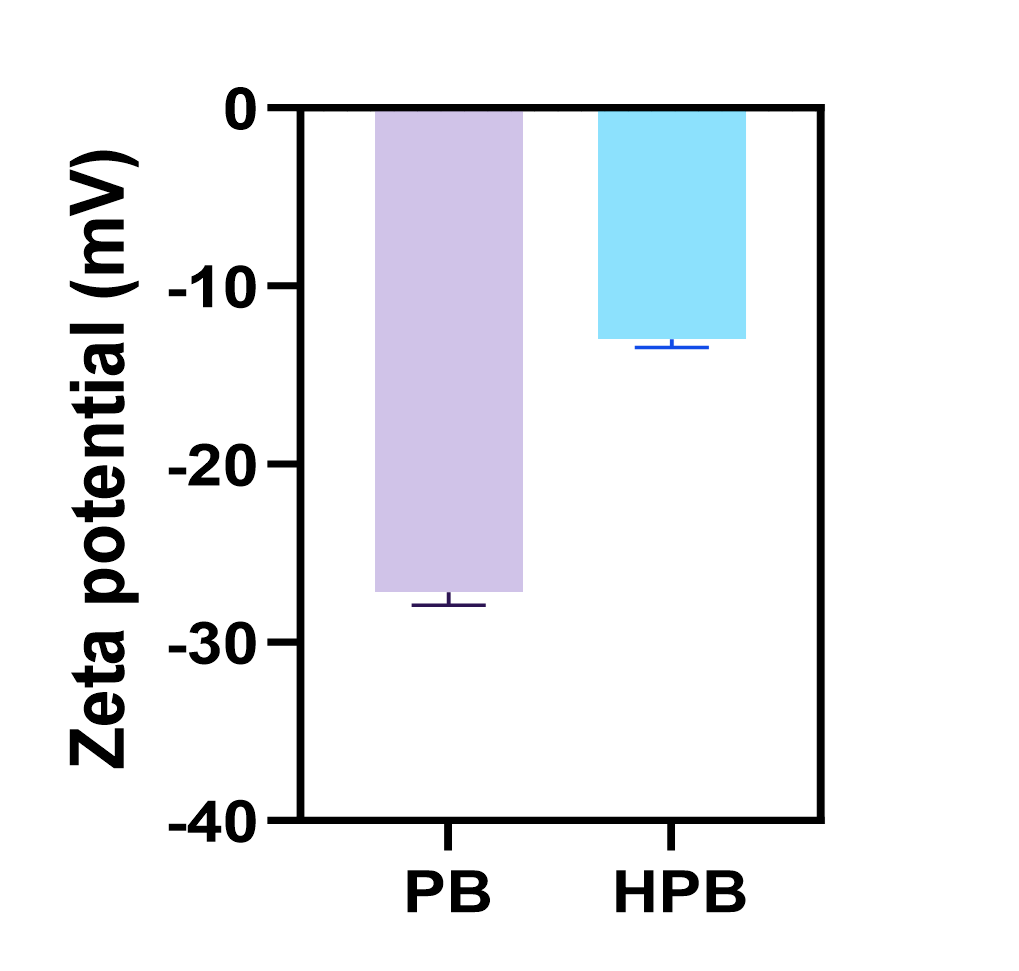


**Figure S3.** Zeta potential of PB and HPB.


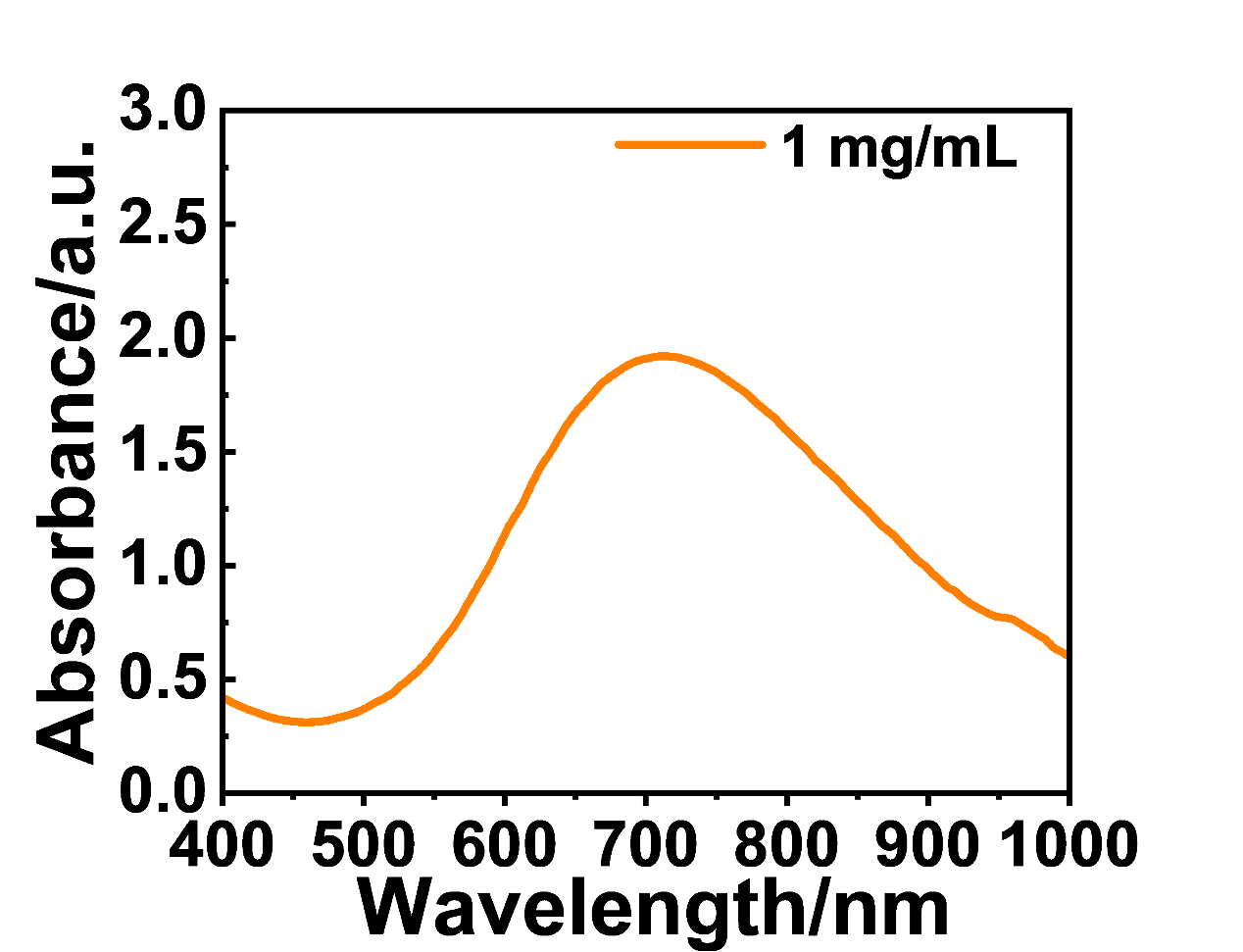


**Figure S4.** UV-Vis absorption spectra of HPB.


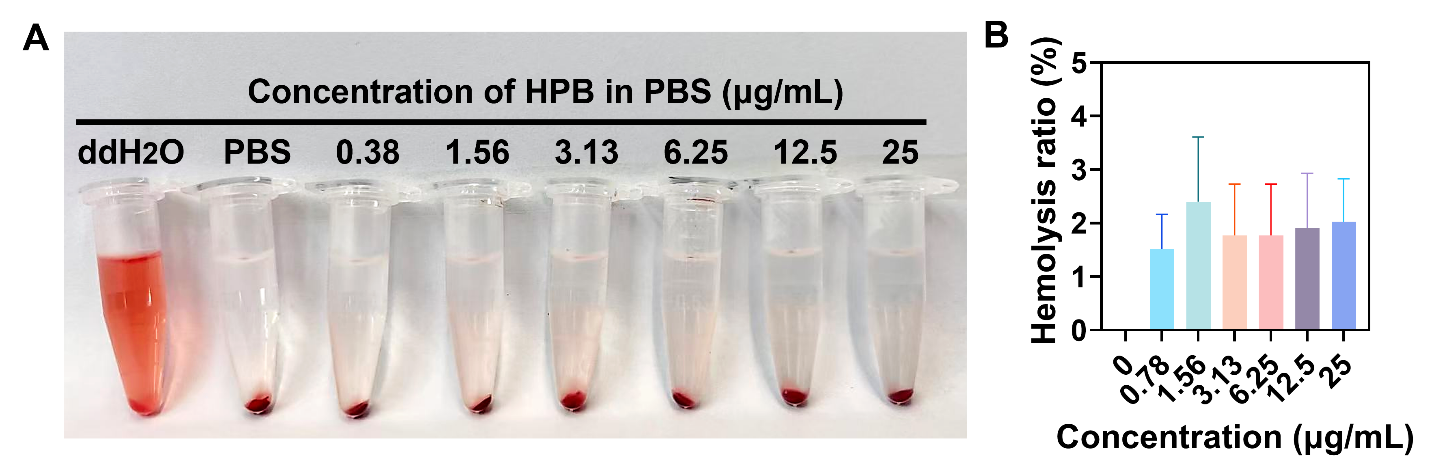


**Figure S5. The hemolysis experiment results of HPB.** (A) The hemolysis of different concentrations of HPB (0.38~25 μg/mL); PBS serves as the negative control; ddH_2_O represents the positive control. (B) Quantitative analysis of the hemolysis rate.


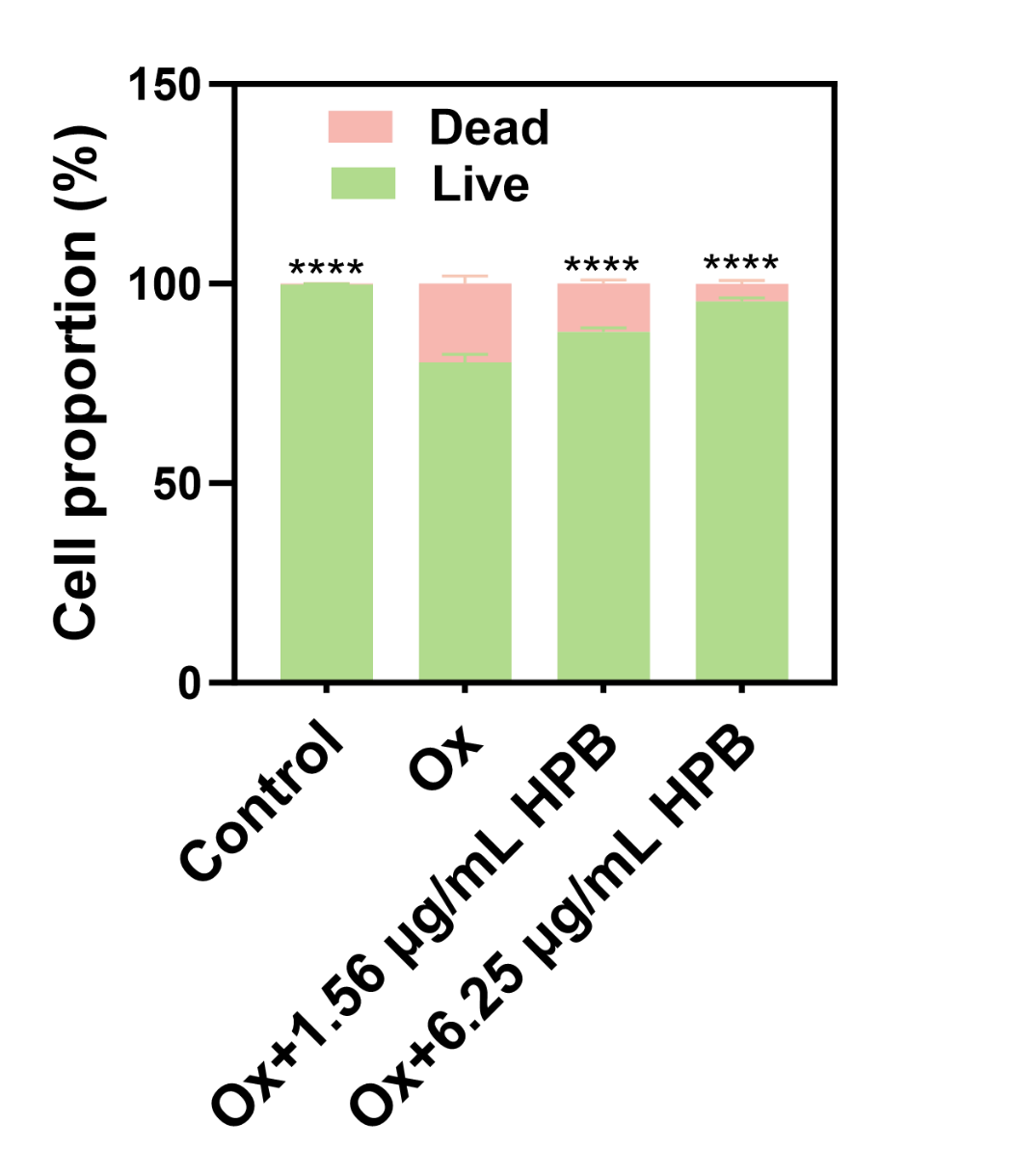


**Figure S6.** Quantitative fluorescence results of Calcein-AM/PI staining. Red color stands for the proportion of dead cells while the green color stands for live cell. **** indicates *P* < 0.0001.


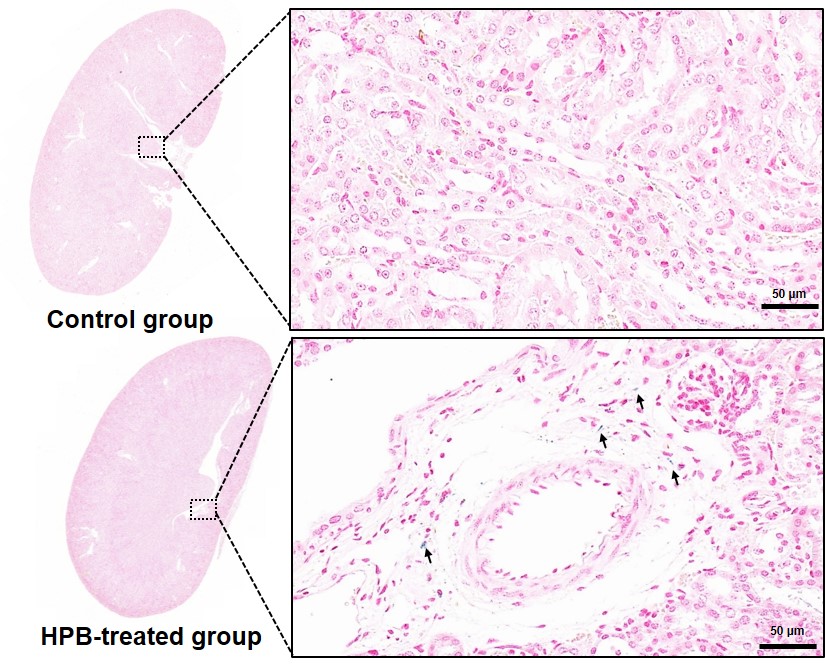


**Figure S7.** The Prussian blue staining image illustrates the distribution of iron ions released from the decomposition of HPB in the kidneys of mice between the control group and the HPB-treated group.


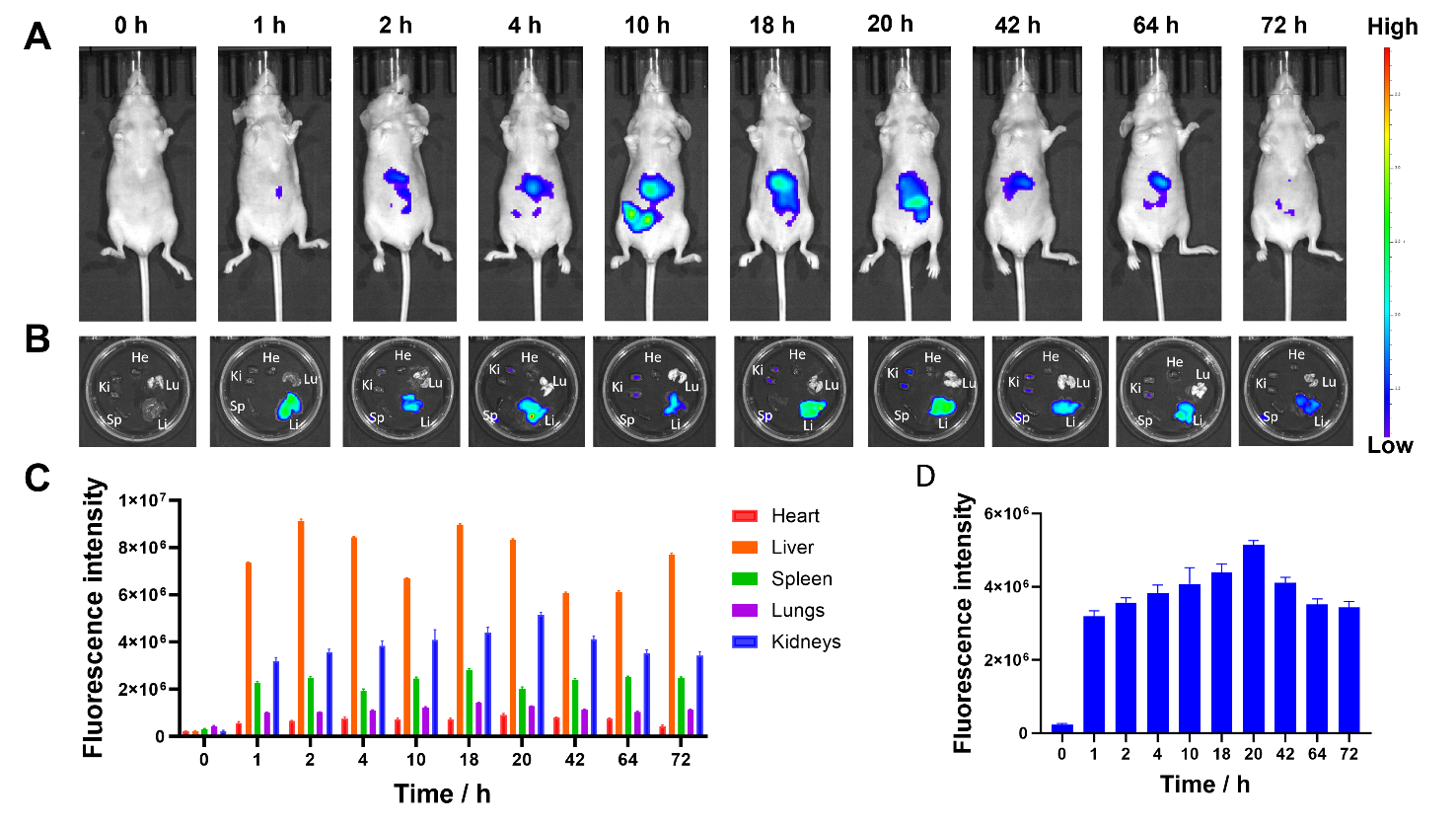


**Figure S8. The biological distribution of HPB in mice.** (A) Fluorescence imaging of HPB–ICG in mice at different time points. (B) Distribution of HPB–ICG in the heart, liver, spleen, lungs, and kidneys of mice at different time points (0, 1, 2, 4, 10, 18, 20, 42, 64 and 72 h). (C) Quantitative statistical analysis of fluorescence intensity in different organs of mice. (D) Quantitative statistical analysis of fluorescence intensity in mice at different time points.


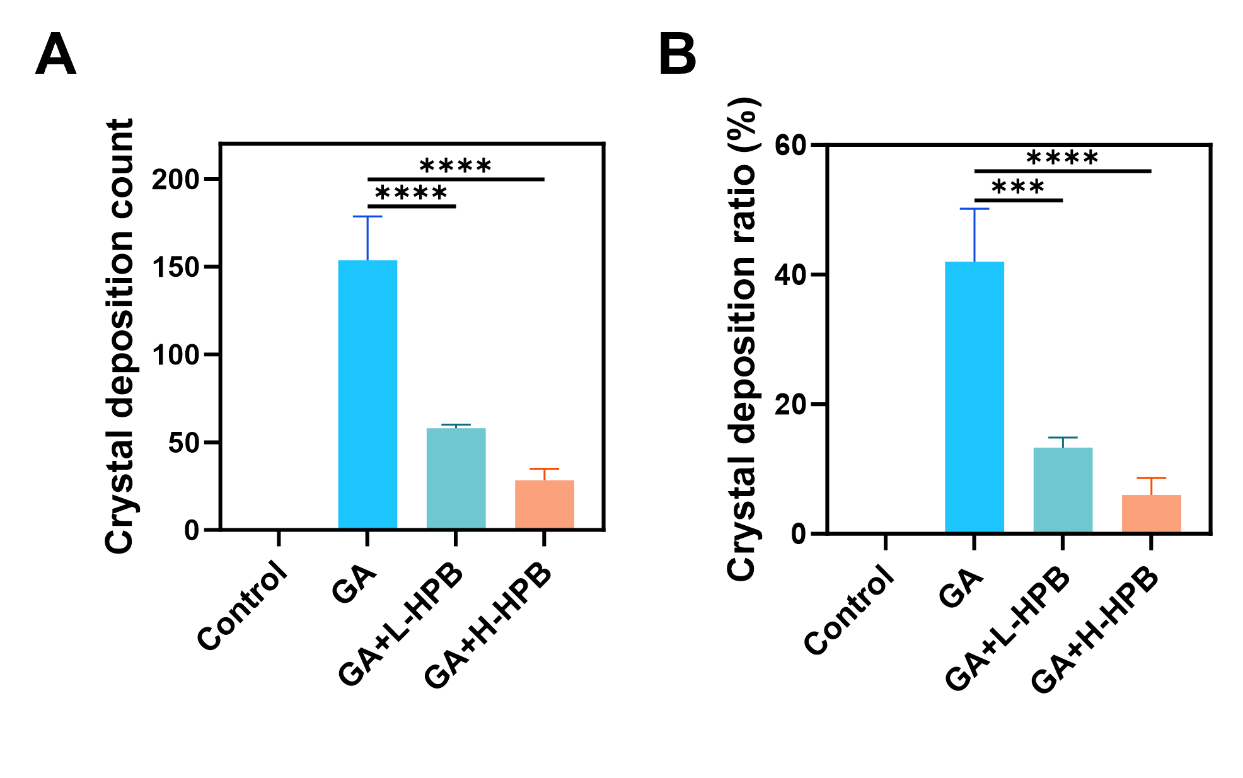


**Figure S9.** Statistical analysis of crystals deposited in kidneys. (A) The count of CaOx crystals deposited. (B) The proportion of crystal distribution in the kidney. *** stands for p < 0.001, **** stands for p < 0.0001. The results above were analyzed by WZ camera.

**Table S1. Antibodies used in immunohistochemistry**

| **Proteins** | **Antibodies** | **Dilution ratio** |
| --- | --- | --- |
| CAT | 66765-1-IG (Proteintech) | 1:300 |
| SOD | 14316-1-AP (Proteintech) | 1:100 |
| Kim-1 | 30948-1-AP (Proteintech) | 1:200 |
| OPN | PB0589 (Boster) | 1:100 |
| CD44 | 60224-1-IG (Proteintech) | 1:300 |
